# *N*-Benzyl-2-(2-bromo­phen­yl)-2-(2-nitro­phen­oxy)acetamide

**DOI:** 10.1107/S1600536810014996

**Published:** 2010-05-08

**Authors:** Huo Ming Li, Jin-Long Wu

**Affiliations:** a Laboratory of Asymmetric Catalysis and Synthesis, Department of Chemistry, Zhejiang University, Hangzhou, Zhejiang 310027, People’s Republic of China

## Abstract

The title compound, C_21_H_17_BrN_2_O_4_, a 2-phen­oxy-2-phenyl­acetamide derivative, exhibits a stereogenic center but crystallizes as a racemate as indicated by the centrosymmetric space group. In the mol­ecular structure, the nitro-substituted benzene ring is coplanar [dihedral angle = 12.9 (1)°] with the plane formed by H—N—C(=O)—C=O due to intra­molecular N—H⋯O hydrogen-bond inter­actions.

## Related literature

For the synthesis and biological activity of 2-phen­oxy-2-phenyl-acetamides, see: Dorsch *et al.* (2002 ▶); Wang *et al.* (2010 ▶); Lau *et al.* (2003 ▶). For additional synthetic procedures, see: Dai & Li (2007 ▶). 
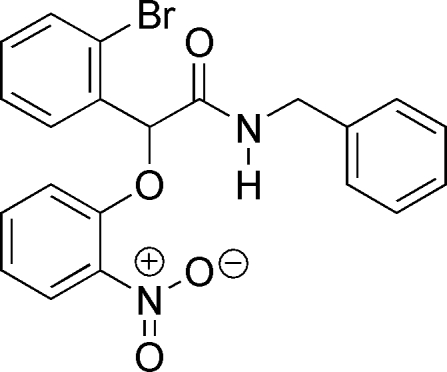


## Experimental

### 

#### Crystal data

C_21_H_17_BrN_2_O_4_
*M* *_r_* = 441.28
Triclinic, 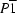

*a* = 7.5818 (5) Å
*b* = 10.4650 (7) Å
*c* = 13.1095 (10) Å
α = 73.939 (6)°
β = 82.878 (6)°
γ = 74.447 (6)°
*V* = 961.56 (12) Å^3^
*Z* = 2
Cu *K*α radiation
μ = 3.17 mm^−1^
*T* = 293 K
0.38 × 0.26 × 0.18 mm

#### Data collection

Oxford Diffraction Xcalibur Atlas Gemini ultra diffractometer
Absorption correction: multi-scan (*CrysAlis PRO*; Oxford Diffraction, 2009 ▶) *T*_min_ = 0.427, *T*_max_ = 0.565
7135 measured reflections
3363 independent reflections
2522 reflections with *I* > 2σ(*I*)
*R*_int_ = 0.030

#### Refinement

*R*[*F* ^2^ > 2σ(*F* ^2^)] = 0.038
*wR*(*F* ^2^) = 0.105
*S* = 1.03
3363 reflections
254 parameters
H-atom parameters constrained
Δρ_max_ = 0.37 e Å^−3^
Δρ_min_ = −0.39 e Å^−3^

### 

Data collection: *CrysAlis PRO* (Oxford Diffraction, 2009 ▶); cell refinement: *CrysAlis PRO*; data reduction: *CrysAlis PRO*; program(s) used to solve structure: *SHELXS97* (Sheldrick, 2008 ▶); program(s) used to refine structure: *SHELXL97* (Sheldrick, 2008 ▶); molecular graphics: *OLEX2* (Dolomanov *et al.*, 2009 ▶); software used to prepare material for publication: *OLEX2*.

## Supplementary Material

Crystal structure: contains datablocks global, I. DOI: 10.1107/S1600536810014996/im2196sup1.cif

Structure factors: contains datablocks I. DOI: 10.1107/S1600536810014996/im2196Isup2.hkl

Additional supplementary materials:  crystallographic information; 3D view; checkCIF report

## Figures and Tables

**Table 1:** Hydrogen-bond geometry (Å, °)

*D*—H⋯*A*	*D*—H	H⋯*A*	*D*⋯*A*	*D*—H⋯*A*
N22—H22⋯O2	0.86	2.08	2.521 (3)	111
N22—H22⋯O25	0.86	2.39	3.227 (4)	164

**Fig. 1.**
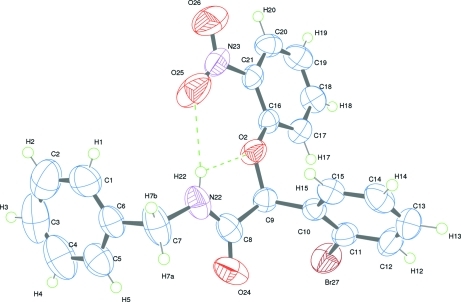
Molecular structure of (1). Displacement ellipsoids are drawn at the 40% probability level and H atoms are shown as small circles of arbitary radii.

## supplementary crystallographic information

### Comment

The title compound, C_21_H_17_BrN_2_O_4_, is a 2-phenoxy-2-phenyl-acetamide derivative, which have been reported to deliver various biological activities such as acting as inhibitors of the coagulation factors Xa and IXa and are therefore used for the therapy of thromboembolic disorder and as safe and effective anticoagulants for myocardial infarction and ischemic disease (Dorsch, *et al.*; 2002, Wang *et al.*, 2010). They are also active as glucokinase activators rendering their use for the treatment of type I and type II diabetes (Lau, *et al.*; 2003).

The title compound has recently been obtained during the Lewis base-catalyzed phenol-Passerini three-component reaction (phenol-P-3CR) from nitrophenols, aryl aldehydes and alkyl isocyanides (Dai & Li; 2007). We report here its crystal structure. In the molecular structure (Fig. 1), there is one benzyl group linked to the amide nitrogen atom. In addition, one 2-bromobenzene and a 2-nitrophenoxy substituents are attached to the α-carbon. The nitro-substituted benzene moiety is coplanar with the plane formed by atoms H22-N22-C8-C9-O2 due to intramolecular hydrogen bond interactions between N22-H22···O2 and N22-H22···O25.

### Experimental

To a solution of 2-nitrophenol (28 mg, 0.20 mmol) in anhydrous MeCN (0.2 mL, 1.0 M) in a 2-mL tube were added 2-bromobenzaldehyde (48.0 mg, 0.26 mmol), *N,N*-diisopropylethylamine (0.004 mL, 0.02 mmol, 10 mol%), and benzyl isocyanide (0.037 mL, 0.30 mmol, 1.5 equiv). The resulting mixture was stirred at 352 K under a nitrogen atmosphere for 72 h. The reaction mixture was concentrated under reduced pressure and the residue was then puried by flash column chromatography (silica gel, eluted with 20% EtOAc in light petroleum ether) to afford the title compound as a yellow solid (84.0 mg, 95%). mp 418-419 K (EtOAc-hexane). Single crystals suitable for X-ray diffraction of the title compound were grown in the mixed solvent of EtOAc and hexane (v:v = 1:3) at 283 K.

### Refinement

The H atoms were placed in calculated positions with C—H = 0.93-0.98 Å, and included in the refinement in riding model, with U_iso_(H) = 1.2U_eq_ (carrier atom).

### Crystal data

**Table:**

C_21_H_17_BrN_2_O_4_	*Z* = 2
*M**_r_* = 441.28	*F*(000) = 448
Triclinic, *P*1	*D*_x_ = 1.524 Mg m^−^^3^
Hall symbol: -P 1	Cu *K*α radiation, λ = 1.54184 Å
*a* = 7.5818 (5) Å	Cell parameters from 3052 reflections
*b* = 10.4650 (7) Å	θ = 3.5–66.9°
*c* = 13.1095 (10) Å	µ = 3.17 mm^−^^1^
α = 73.939 (6)°	*T* = 293 K
β = 82.878 (6)°	Block, colorless
γ = 74.447 (6)°	0.38 × 0.26 × 0.18 mm
*V* = 961.56 (12) Å^3^	

### Data collection

**Table:**

Oxford Diffraction Xcalibur Atlas Gemini ultra diffractometer	3363 independent reflections
Radiation source: fine-focus sealed tube	2522 reflections with *I* > 2σ(*I*)
graphite	*R*_int_ = 0.030
Detector resolution: 10.3592 pixels mm^-1^	θ_max_ = 66.6°, θ_min_ = 3.5°
ω scans	*h* = −9→8
Absorption correction: multi-scan (*CrysAlis PRO*; Oxford Diffraction, 2009)	*k* = −11→12
*T*_min_ = 0.427, *T*_max_ = 0.565	*l* = −15→15
7135 measured reflections	

### Refinement

**Table:**

Refinement on *F*^2^	Secondary atom site location: difference Fourier map
Least-squares matrix: full	Hydrogen site location: inferred from neighbouring sites
*R*[*F*^2^ > 2σ(*F*^2^)] = 0.038	H-atom parameters constrained
*wR*(*F*^2^) = 0.105	*w* = 1/[σ^2^(*F*_o_^2^) + (0.0523*P*)^2^ + 0.350*P*] where *P* = (*F*_o_^2^ + 2*F*_c_^2^)/3
*S* = 1.03	(Δ/σ)_max_ < 0.001
3363 reflections	Δρ_max_ = 0.37 e Å^−^^3^
254 parameters	Δρ_min_ = −0.39 e Å^−^^3^
0 restraints	Extinction correction: *SHELXL97* (Sheldrick, 2008), Fc^*^=kFc[1+0.001xFc^2^λ^3^/sin(2θ)]^-1/4^
Primary atom site location: structure-invariant direct methods	Extinction coefficient: 0.0258 (10)

### Special details

**Table:**

Experimental. (CrysAlis Pro; Oxford Diffraction, 2009) Version 1.171.33.53 (release 17-11-2009 CrysAlis171 .NET) (compiled Nov 17 2009,16:58:22) Empirical absorption correction using spherical harmonics, implemented in SCALE3 ABSPACK scaling algorithm.
Geometry. All esds (except the esd in the dihedral angle between two l.s. planes) are estimated using the full covariance matrix. The cell esds are taken into account individually in the estimation of esds in distances, angles and torsion angles; correlations between esds in cell parameters are only used when they are defined by crystal symmetry. An approximate (isotropic) treatment of cell esds is used for estimating esds involving l.s. planes.
Refinement. Refinement of *F*^2^ against ALL reflections. The weighted *R*-factor wR and goodness of fit *S* are based on *F*^2^, conventional *R*-factors *R* are based on *F*, with *F* set to zero for negative *F*^2^. The threshold expression of *F*^2^ > σ(*F*^2^) is used only for calculating *R*-factors(gt) etc. and is not relevant to the choice of reflections for refinement. *R*-factors based on *F*^2^ are statistically about twice as large as those based on *F*, and *R*- factors based on ALL data will be even larger.

### Fractional atomic coordinates and isotropic or equivalent isotropic displacement parameters (Å^2^)

**Table:**

	*x*	*y*	*z*	*U*_iso_*/*U*_eq_	
Br27	0.67572 (5)	0.56595 (4)	0.87665 (3)	0.0848 (2)	
O2	0.3651 (3)	0.99683 (19)	0.82814 (14)	0.0599 (5)	
O24	0.6787 (3)	0.8846 (3)	0.63093 (18)	0.0856 (7)	
O25	0.2519 (5)	1.2538 (2)	0.8109 (2)	0.1010 (9)	
O26	0.0566 (5)	1.3176 (3)	0.9264 (3)	0.1318 (13)	
N22	0.5004 (4)	1.0913 (3)	0.64616 (19)	0.0712 (7)	
H22	0.4153	1.1318	0.6846	0.085*	
N23	0.1698 (4)	1.2288 (3)	0.8964 (2)	0.0653 (7)	
C1	0.6438 (6)	1.3641 (4)	0.5979 (3)	0.0878 (11)	
H1	0.5190	1.4028	0.6064	0.105*	
C2	0.7724 (10)	1.4280 (5)	0.6178 (3)	0.1174 (18)	
H2	0.7341	1.5090	0.6395	0.141*	
C3	0.9541 (11)	1.3690 (8)	0.6046 (4)	0.126 (2)	
H3	1.0401	1.4101	0.6182	0.152*	
C4	1.0118 (7)	1.2529 (7)	0.5724 (4)	0.1164 (18)	
H4	1.1365	1.2150	0.5628	0.140*	
C5	0.8863 (5)	1.1907 (4)	0.5537 (3)	0.0829 (10)	
H5	0.9267	1.1098	0.5320	0.099*	
C6	0.7016 (4)	1.2457 (4)	0.5664 (2)	0.0629 (8)	
C7	0.5676 (6)	1.1746 (5)	0.5464 (3)	0.0911 (13)	
H7B	0.4646	1.2424	0.5108	0.109*	
H7A	0.6259	1.1159	0.4998	0.109*	
C8	0.5655 (4)	0.9562 (4)	0.6794 (2)	0.0615 (8)	
C9	0.4855 (4)	0.8876 (3)	0.7880 (2)	0.0515 (6)	
H9	0.5849	0.8396	0.8363	0.062*	
C10	0.3835 (4)	0.7874 (3)	0.7772 (2)	0.0477 (6)	
C11	0.4463 (4)	0.6461 (3)	0.8127 (2)	0.0577 (7)	
C12	0.3448 (5)	0.5585 (3)	0.8030 (3)	0.0745 (9)	
H12	0.3896	0.4642	0.8271	0.089*	
C13	0.1799 (5)	0.6103 (4)	0.7584 (3)	0.0750 (9)	
H13	0.1110	0.5511	0.7532	0.090*	
C14	0.1141 (4)	0.7491 (4)	0.7209 (2)	0.0673 (8)	
H14	0.0017	0.7842	0.6895	0.081*	
C15	0.2163 (4)	0.8368 (3)	0.7301 (2)	0.0558 (7)	
H15	0.1716	0.9309	0.7040	0.067*	
C16	0.3115 (4)	0.9775 (3)	0.93255 (19)	0.0460 (6)	
C17	0.3463 (4)	0.8503 (3)	1.0060 (2)	0.0544 (7)	
H17	0.4134	0.7728	0.9846	0.065*	
C18	0.2821 (4)	0.8388 (3)	1.1098 (2)	0.0631 (8)	
H18	0.3077	0.7534	1.1583	0.076*	
C19	0.1805 (4)	0.9514 (4)	1.1434 (2)	0.0698 (9)	
H19	0.1373	0.9420	1.2140	0.084*	
C20	0.1435 (4)	1.0772 (3)	1.0723 (2)	0.0628 (8)	
H20	0.0739	1.1537	1.0943	0.075*	
C21	0.2096 (4)	1.0908 (3)	0.9674 (2)	0.0488 (6)	

### Atomic displacement parameters (Å^2^)

**Table:**

	*U*^11^	*U*^22^	*U*^33^	*U*^12^	*U*^13^	*U*^23^
Br27	0.0661 (3)	0.0668 (3)	0.1161 (4)	0.00496 (17)	−0.0286 (2)	−0.0247 (2)
O2	0.0875 (14)	0.0447 (10)	0.0454 (10)	−0.0160 (10)	0.0037 (9)	−0.0113 (8)
O24	0.0676 (14)	0.124 (2)	0.0680 (14)	−0.0247 (14)	0.0139 (12)	−0.0354 (14)
O25	0.165 (3)	0.0495 (13)	0.0740 (16)	−0.0125 (15)	−0.0038 (18)	−0.0060 (12)
O26	0.135 (3)	0.0565 (16)	0.171 (3)	0.0045 (17)	0.036 (2)	−0.0219 (18)
N22	0.0792 (18)	0.085 (2)	0.0506 (14)	−0.0405 (16)	0.0013 (13)	−0.0016 (13)
N23	0.0701 (16)	0.0466 (14)	0.084 (2)	−0.0138 (13)	−0.0136 (14)	−0.0209 (13)
C1	0.103 (3)	0.087 (3)	0.062 (2)	−0.022 (2)	0.0102 (19)	−0.0068 (19)
C2	0.201 (6)	0.094 (3)	0.068 (3)	−0.065 (4)	0.000 (3)	−0.015 (2)
C3	0.175 (6)	0.149 (5)	0.083 (3)	−0.113 (5)	−0.031 (4)	0.009 (3)
C4	0.085 (3)	0.156 (5)	0.099 (3)	−0.057 (3)	−0.020 (2)	0.014 (3)
C5	0.081 (2)	0.089 (3)	0.069 (2)	−0.025 (2)	−0.0016 (18)	−0.0021 (18)
C6	0.071 (2)	0.078 (2)	0.0367 (14)	−0.0313 (17)	−0.0043 (13)	0.0037 (14)
C7	0.104 (3)	0.126 (3)	0.0500 (18)	−0.070 (3)	−0.0088 (18)	0.0084 (19)
C8	0.0524 (17)	0.088 (2)	0.0523 (17)	−0.0299 (17)	−0.0051 (14)	−0.0172 (16)
C9	0.0577 (16)	0.0545 (15)	0.0455 (14)	−0.0160 (13)	−0.0042 (12)	−0.0152 (12)
C10	0.0467 (14)	0.0546 (15)	0.0455 (14)	−0.0130 (12)	0.0004 (11)	−0.0192 (12)
C11	0.0501 (15)	0.0574 (17)	0.0684 (18)	−0.0065 (13)	−0.0058 (13)	−0.0256 (14)
C12	0.077 (2)	0.0560 (18)	0.100 (3)	−0.0164 (16)	−0.0095 (19)	−0.0330 (17)
C13	0.075 (2)	0.079 (2)	0.088 (2)	−0.0344 (19)	−0.0033 (18)	−0.0341 (19)
C14	0.0490 (16)	0.098 (3)	0.0607 (18)	−0.0223 (17)	−0.0044 (14)	−0.0242 (17)
C15	0.0513 (15)	0.0632 (17)	0.0507 (15)	−0.0112 (14)	−0.0016 (12)	−0.0142 (13)
C16	0.0512 (14)	0.0500 (14)	0.0443 (14)	−0.0208 (12)	−0.0049 (11)	−0.0148 (11)
C17	0.0654 (17)	0.0504 (15)	0.0479 (15)	−0.0143 (13)	−0.0077 (13)	−0.0114 (12)
C18	0.0674 (19)	0.071 (2)	0.0466 (16)	−0.0188 (16)	−0.0079 (14)	−0.0045 (14)
C19	0.0681 (19)	0.094 (3)	0.0481 (16)	−0.0191 (18)	−0.0003 (14)	−0.0220 (17)
C20	0.0556 (17)	0.075 (2)	0.0663 (19)	−0.0107 (15)	−0.0044 (14)	−0.0361 (16)
C21	0.0486 (14)	0.0482 (14)	0.0561 (16)	−0.0161 (12)	−0.0107 (12)	−0.0165 (12)

### Geometric parameters (Å, °)

**Table:**

Br27—C11	1.903 (3)	C8—C9	1.538 (4)
O2—C16	1.354 (3)	C9—C10	1.504 (4)
O2—C9	1.439 (3)	C9—H9	0.9800
O24—C8	1.218 (4)	C10—C15	1.386 (4)
O25—N23	1.209 (4)	C10—C11	1.388 (4)
O26—N23	1.203 (4)	C11—C12	1.384 (4)
N22—C8	1.331 (4)	C12—C13	1.357 (5)
N22—C7	1.473 (4)	C12—H12	0.9300
N22—H22	0.8600	C13—C14	1.370 (5)
N23—C21	1.461 (4)	C13—H13	0.9300
C1—C6	1.358 (5)	C14—C15	1.387 (4)
C1—C2	1.406 (7)	C14—H14	0.9300
C1—H1	0.9300	C15—H15	0.9300
C2—C3	1.361 (8)	C16—C17	1.391 (4)
C2—H2	0.9300	C16—C21	1.391 (4)
C3—C4	1.341 (8)	C17—C18	1.371 (4)
C3—H3	0.9300	C17—H17	0.9300
C4—C5	1.367 (6)	C18—C19	1.375 (5)
C4—H4	0.9300	C18—H18	0.9300
C5—C6	1.372 (5)	C19—C20	1.367 (5)
C5—H5	0.9300	C19—H19	0.9300
C6—C7	1.494 (5)	C20—C21	1.386 (4)
C7—H7B	0.9700	C20—H20	0.9300
C7—H7A	0.9700		
			
C16—O2—C9	121.1 (2)	C10—C9—H9	109.5
C8—N22—C7	122.8 (3)	C8—C9—H9	109.5
C8—N22—H22	118.6	C15—C10—C11	117.2 (3)
C7—N22—H22	118.6	C15—C10—C9	119.1 (3)
O26—N23—O25	120.9 (3)	C11—C10—C9	123.7 (2)
O26—N23—C21	118.1 (3)	C12—C11—C10	121.3 (3)
O25—N23—C21	120.9 (3)	C12—C11—Br27	117.6 (2)
C6—C1—C2	120.0 (4)	C10—C11—Br27	121.0 (2)
C6—C1—H1	120.0	C13—C12—C11	120.1 (3)
C2—C1—H1	120.0	C13—C12—H12	120.0
C3—C2—C1	118.7 (5)	C11—C12—H12	120.0
C3—C2—H2	120.7	C12—C13—C14	120.4 (3)
C1—C2—H2	120.7	C12—C13—H13	119.8
C4—C3—C2	121.5 (5)	C14—C13—H13	119.8
C4—C3—H3	119.3	C13—C14—C15	119.5 (3)
C2—C3—H3	119.3	C13—C14—H14	120.2
C3—C4—C5	119.6 (5)	C15—C14—H14	120.2
C3—C4—H4	120.2	C10—C15—C14	121.5 (3)
C5—C4—H4	120.2	C10—C15—H15	119.3
C4—C5—C6	121.1 (5)	C14—C15—H15	119.3
C4—C5—H5	119.4	O2—C16—C17	123.9 (2)
C6—C5—H5	119.4	O2—C16—C21	117.9 (2)
C1—C6—C5	119.1 (4)	C17—C16—C21	118.2 (2)
C1—C6—C7	121.0 (4)	C18—C17—C16	120.2 (3)
C5—C6—C7	119.9 (4)	C18—C17—H17	119.9
N22—C7—C6	111.5 (3)	C16—C17—H17	119.9
N22—C7—H7B	109.3	C17—C18—C19	121.2 (3)
C6—C7—H7B	109.3	C17—C18—H18	119.4
N22—C7—H7A	109.3	C19—C18—H18	119.4
C6—C7—H7A	109.3	C20—C19—C18	119.5 (3)
H7B—C7—H7A	108.0	C20—C19—H19	120.2
O24—C8—N22	125.8 (3)	C18—C19—H19	120.2
O24—C8—C9	118.7 (3)	C19—C20—C21	120.0 (3)
N22—C8—C9	115.5 (3)	C19—C20—H20	120.0
O2—C9—C10	111.1 (2)	C21—C20—H20	120.0
O2—C9—C8	106.1 (2)	C20—C21—C16	120.9 (3)
C10—C9—C8	111.0 (2)	C20—C21—N23	117.2 (3)
O2—C9—H9	109.5	C16—C21—N23	121.9 (2)

### Hydrogen-bond geometry (Å, °)

**Table:**

*D*—H···*A*	*D*—H	H···*A*	*D*···*A*	*D*—H···*A*
N22—H22···O2	0.86	2.08	2.521 (3)	111
N22—H22···O25	0.86	2.39	3.227 (4)	164
